# Intra-Articular Injection of (−)-Epigallocatechin 3-Gallate (EGCG) Ameliorates Cartilage Degeneration in Guinea Pigs with Spontaneous Osteoarthritis

**DOI:** 10.3390/antiox10020178

**Published:** 2021-01-26

**Authors:** Hsuan-Ti Huang, Tsung-Lin Cheng, Chung-Da Yang, Chi-Fen Chang, Cheng-Jung Ho, Shu-Chun Chuang, Jhong-You Li, Shih-Hao Huang, Yi-Shan Lin, Hsin-Yi Shen, Tsung-Han Yu, Lin Kang, Sung-Yen Lin, Chung-Hwan Chen

**Affiliations:** 1 Orthopaedic Research Center, Kaohsiung Medical University, Kaohsiung 80701, Taiwan; hthuang@kmu.edu.tw (H.-T.H.); junglecc@kmu.edu.tw (T.-L.C.); u105801010@kmu.edu.tw (C.-J.H.); f86225016@ntu.edu.tw (S.-C.C.); u109508016@kmu.edu.tw (J.-Y.L.); u108508017@kmu.edu.tw (S.-H.H.); r950084@kmu.edu.tw (Y.-S.L.); u106000049@kmu.edu.tw (H.-Y.S.); u101001078@kmu.edu.tw (T.-H.Y.); 2 Department of Orthopedics, Kaohsiung Medical University Hospital, Kaohsiung Medical University, Kaohsiung 80701, Taiwan; 3 Regeneration Medicine and Cell Therapy Research Center, Kaohsiung Medical University, Kaohsiung 80701, Taiwan; 4 Departments of Orthopedics, College of Medicine, Kaohsiung Medical University, Kaohsiung 80701, Taiwan; 5 Department of Orthopedics, Kaohsiung Municipal Ta-Tung Hospital, Kaohsiung 80145, Taiwan; 6 Musculoskeletal Regeneration Research Center, Kaohsiung Medical University, Kaohsiung 80701, Taiwan; 7 Department of Physiology, College of Medicine, Kaohsiung Medical University, Kaohsiung 80701, Taiwan; 8 Graduate Institute of Animal Vaccine Technology, College of Veterinary Medicine, National Pingtung University of Science and Technology, Pingtung 91201, Taiwan; cdyang@mail.npust.edu.tw; 9 Department of Anatomy, School of Medicine, China Medical University, Taichung 40402, Taiwan; cfchang@mail.cmu.edu.tw; 10 Department of Orthopedics, Kaohsiung Municipal Hsiao-Kang Hospital, Kaohsiung Medical University, Kaohsiung 80701, Taiwan; 11 Department of Obstetrics and Gynecology, National Cheng Kung University Hospital, College of Medicine, National Cheng Kung University, Tainan 70457, Taiwan; 12 Department of Healthcare Administration and Medical Informatics, Kaohsiung Medical University, Kaohsiung 80701, Taiwan; 13 Institute of Medical Science and Technology, National Sun Yat-Sen University, Kaohsiung 80424, Taiwan

**Keywords:** (−)-epigallocatechin 3-gallate (EGCG), cartilage, cell senescence, guinea pigs, osteoarthritis, senescence

## Abstract

Osteoarthritis (OA) is the most prevalent joint disease that causes an enormous burden of disease worldwide. (−)-Epigallocatechin 3-gallate (EGCG) has been reported to reduce post-traumatic OA progression through its anti-inflammatory property. Aging is the most crucial risk factor of OA, and the majority of OA incidences are related to age and not trauma. In this study, we assess whether EGCG can ameliorate cartilage degradation in primary OA. In an in-vitro study, real-time PCR was performed to assess the expression of genes associated with human articular chondrocyte homeostasis. A spontaneously occurring OA model in guinea pigs was used to investigate the effect of EGCG in vivo. OA severity was evaluated using Safranin O staining and Osteoarthritis Research Society International (OARSI) scores, as well as by immunohistochemical (IHC) analysis to determine the protein level of type II collagen (Col II), matrix metalloproteinase 13 (MMP-13), and p16 ^ink4a^ in articular cartilage. In the in-vitro study, EGCG increased the gene expression of aggrecan and Col II and decreased the expression of interleukin-1, cyclooxygenase 2, MMP-13, alkaline phosphatase, Col X, and p16 ^Ink4a^; EGCG treatment also attenuated the degraded cartilage with a lower OARSI score. Meanwhile, IHC results showed that EGCG exerted an anti-OA effect by reducing ECM degradation, cartilage inflammation, and cell senescence with a less-immunostained Col II, MMP-13, and p16 ^Ink4a^. In conclusion, these findings suggest that EGCG may be a potential disease-modifying OA drug for the treatment of primary OA.

## 1. Introduction

Osteoarthritis (OA) is an age-related disease that is considered to be the leading cause of functional disability in the older population [1]. It is recognized as a chronic degenerative joint disease, with cartilage degradation with increasing age. Age is the most significant risk factor for OA, but it does not directly induce OA. Aging changes the joint structure, leading to vulnerability to injury; it also decreases the anabolic activity and increases the catabolic activity of chondrocytes, resulting in progressive cartilage degradation [1,2,3,4]. These aging-related changes in chondrocytes decrease their ability to maintain hemostasis in response to stress, causing gradual cartilage wear.

(−)-Epigallocatechin 3-gallate (EGCG), the major bioactive component of green tea catechins, has gained interest in OA research because of its potent anti-inflammatory and antioxidative properties [5,6,7,8,9,10,11,12,13,14,15,16,17,18,19,20,21]. The chondroprotective effect of EGCG has been widely investigated using human articular chondrocytes [22,23,24], animal articular chondrocytes [25,26], bovine cartilage explants [27], and surgically induced OA animal models [28,29,30]. These studies have demonstrated that EGCG could mitigate OA progression by inhibiting the expression of proinflammatory genes (i.e., cyclooxygenase 2 (COX-2), matrix metalloproteinase-1(MMP-1), MMP-3, MMP-13, inducible nitric oxide synthase, tumor necrosis factor-α (TNF-α), transforming growth factor-β2, a disintegrin and metalloproteinase with thrombospondin motifs (ADAMTS)-5, aggrecanase-1, -2) [22,23,26,28,31,32,33], reducing the production of nitric oxide [22,34] and prostaglandin E2 [32,35], as well as increasing chondrogenic regeneration genes (i.e., aggrecan, collagen type II (Col II), and SOX9) [25,28].

Cell senescence is a phenomenon of irreversible cell cycle arrest that is closely related to the age-related excess of oxidative stress. The excess reactive oxygen species by increasing age induces DNA damage and promotes cell senescence [36]. Proinflammatory mediators also increased with age through cell senescence [37]. Cell senescence and the development of the senescence-associated secretory phenotype increase the progression of joint inflammation and OA development [38,39]. Cell senescence compromises the ability of chondrocytes to maintain the hemostasis of cartilage, which is considered a significant factor in the pathogenesis of OA [40]. Previous studies on the treatment of OA by EGCG mainly focused on the anti-inflammatory response, and the effect of EGCG on cell senescence remains unclear [24].

To get a better understanding of the pathophysiology and to assist with the development of new treating agents, a number of animal models for OA have been developed. There are three main types of animal studies for in-vivo OA models: (1) a naturally occurring primary OA model, (2) a secondary OA model that includes surgical- or trauma-induced cartilage degradation, and (3) intra-articular injections of chondrotoxic or proinflammatory substances [41]. The best animal models for OA research remain inconclusive. Surgically induced OA models are representative of post-traumatic OA, but most OA cases are not secondary to trauma. Chemical-intervention-induced cartilage degradation triggers an acute episode of chondrocyte death, extracellular matrix (ECM) loss, and joint inflammation, which do not usually occur in primary OA [42,43]. Dunkin–Hartley (DH) guinea pigs develop OA spontaneously in as early as three months of age and are proposed as a practical model for investigating the progression of primary OA [44]. The disease pathogenesis of the naturally occurring guinea pig OA model is similar to that seen in humans, and it can be used to investigate the risk factors for OA, such as age and obesity [45]. Furthermore, previous studies have also demonstrated that the OA histopathology is similar between the naturally occurring OA guinea pig model and the disease in humans [46]. We previously found the intra-articular injections of EGCG ameliorated OA in the post-traumatic animal model by attenuating the inflammation on synovial tissue and cartilage with subsequently decreasing matrix degradation. We further note EGCG may modulate chondrocyte apoptosis by the activation of cytoprotective autophagy [47]. We, therefore, hypothesize that EGCG may attenuate the progression of cartilage degeneration in aging-related OA. This present study aims to investigate the antiosteoarthritic effects of EGCG in human articular chondrocytes using an aging-related OA model in guinea pigs.

## 2. Materials and Methods

### 2.1. Cell Culture

The study used Clonetics^TM^ normal human articular chondrocytes-knee (NHAC-kn) (Lonza, Walkersville, MD, USA). The chondrocytes were cultured in the low glucose Dulbecco’s modified Eagle’s medium (DMEM) mixed with 10% fetal bovine serum (FBS), 1.5 g/L sodium bicarbonate, 1% nonessential amino acid (NEAA), 1% insulin–transferrin–selenium, and 1% penicillin and streptomycin at 37 °C in a humidified atmosphere of 5% CO_2_ and 95% air. NHAC-kn was induced by 10 ng/mL interleukin (IL)-1β for 48 h and then treated with 10 μM EGCG for 24 or 72 h.

### 2.2. Cell Viability Assessment

In general, cell proliferation is regarded as the measurement of cytotoxicity via the 3-(4,5-dimethylthiazol-2-yl)-5-(3-carboxymethoxyphenyl)-2-(4-sulfophenyl)-2H-tetrazolium (MTS) assay. The CellTiter 96 AQueous One Solution cell proliferation assay (Promega, Madison, WI), which is a colorimetric method for determining the number of viable cells in culture, was used to count cell numbers. Briefly, chondrocytes at a density of 5 × 10^3^ cells/well were seeded in 96-well plates. The culture wells were treated with various concentrations of EGCG (1, 10, 20, 50, 200, and 500 μM). Subsequently, the MTS solution with a concentration of 50 μg per 100 μL medium was added to each well. Then, the mixture was incubated for 2 h at 37 °C. Finally, 0.2 mL of the medium, including formazan, was sucked and placed in a new well plate. Optical density was measured at 490 nm using a microplate ELISA reader.

### 2.3. Senescence-Associated Beta-Galactosidase (SAβGal) Activity Analysis

A senescence-associated beta-galactosidase (SAβGal) activity analysis was proposed in 1995 by Dimri et al. They showed that when beta-galactosidase assays were carried out at pH 6.0, only cells in the senescence state developed staining. In the classic assay for senescence cells, SAβGal catalyzes the hydrolysis of X-gal, which results in the accumulation of a distinctive blue color in senescent cells [48,49,50]. Cells were fixed and stained using the senescence β-Galactosidase Staining Kit (9860; Cell Signaling Technology), according to the manufacturer’s protocols. The data were quantified and assessed with Image-Pro Plus 5.1version software, and the results are represented with the ratio of the relative density of the brown-stained area to the total area (density/total area) [51].

### 2.4. Real-Time Quantitative PCR (RT-qPCR)

The expressions of the related gene were analyzed using quantification PCR. The mRNA expression for aggrecan, Col II, MMP-13, IL-1, alkaline phosphatase (ALP), collagen type X (Col X), p16 ^Ink4a^, and COX-2 in HACs was carried out by RT-qPCR. The RNA was isolated using an RNA extraction kit, and cDNA was synthesized using an iScript reverse transcriptase kit (Bio-Rad Laboratories, Hercules, CA, USA). RT-qPCR was performed in duplicate for each sample to determine relative gene expression, using glyceraldehyde 3-phosphate dehydrogenase as a housekeeping control. Relative quantitation of the genes was normalized based on GAPDH content. The PCR primer sequences of the genes of interest used are shown in Table 1.

### 2.5. Animal Experiments

The experimental protocols were conducted according to the “Guide for the Care and Use of Laboratory Animals” of Kaohsiung Medical University and with the approval of the Kaohsiung Medical University Institutional Animal Care and Use Committee (IACUC Approval No. 103160). Twenty-four female DH strain guinea pigs were used in this experiment. Eight animals were euthanized at 6 months of age, and bilateral knee joints were harvest for analysis. The other sixteen DH strain guinea pigs were randomly allocated into two experimental groups that received either intra-articular injections of EGCG (Sigma-Aldrich, St. Louis MO, USA) or the same volume of phosphate-buffered saline (PBS) as control. The EGCG group comprised 8 guinea pigs that received intra-articular injections [47,52,53] of EGCG at a dosage of 10 μM (40 μL) [47] once a week for 12 consecutive weeks from 6 months of age. The same volume of PBS (40 μL) was injected into the right knee joints of controlled animals (*n* = 8). A week after the final injection, all animals were euthanized (at 9 months of age), and bilateral knee joint tissues were collected for further analysis.

### 2.6. Endurance Test

Endurance tests were performed on Columbus Instruments rodent treadmills (Columbus, OH), with a running speed of 35 m/min for 10 min. Animals were familiarized with the endurance test with a training protocol (at a speed of 10 m/min for 15 min). Measurements were performed three times for each animal before and after treatment, and average data were calculated. The test was terminated when the animal could not run anymore or at the maximum time of running endurance [52,53,54].

### 2.7. Gross Observations and Histopathological Analysis

After harvesting, the gross visual appearance of knee joints was examined and recorded with a digital camera. For histopathological analysis, the samples were fixed in 10% buffered formalin for 24 h and then decalcified with 10% formic acid. The samples were then dehydrated, embedded in paraffin blocks, and cut with 5-μm thickness in the coronary plane for staining. Glycosaminoglycan (GAG) was stained with safranin O–Fast Green (1% safranin O, counterstained with 0.75% hematoxylin, and then 1% Fast Green; Sigma) and quantified with Image-Pro Plus 5.0 software (Media Cybernetics, Rockville, MD, USA). The relative density of the red-stained area to the total area (density/total area) in each group was calculated. The severity of OA was assessed using a semiquantitative histopathological grading system according to the Osteoarthritis Research Society International (OARSI), using articular surface integrity, extracellular cellular matrix content, cellularity, and bone remolding. The average scores from two blinded observers were the final observation results for statistical analysis [55,56].

### 2.8. Immunohistochemistry (IHC) for Col II, MMP-13, and Marker of Cellular Senescence (p16 ^Ink4a^)

For Col II and MMP-13 IHC staining, the endogenous peroxidase in tissues was firstly blocked by 3% H_2_O_2_, and then the samples were digested by a mixture of 2.5% hyaluronidase and 1 mg/mL pronate at 37 °C for 1 h for epitope retrieval. The sections were blocked with fetal bovine serum for 1 h and incubated with the monoclonal antibody to Col II (ab34712, Abcam, Cambridge, MA, USA), MMP-13 primary antibodies (ab39012, Abcam, Cambridge, MA, USA), and p16 ^Ink4a^ (ab54210, Abcam, Cambridge, MA, USA) at 37 °C for 4 h. After incubation with a primary antibody, an EXPOSE mouse- and rabbit-specific horseradish peroxidase-diaminobenzidine detection IHC kit (Abcam, Cambridge, MA, USA) was applied. Finally, sections were counterstained with hematoxylin. The data were quantified using Image-Pro Plus version 5.1 software. The sections appearing brown or brownish-yellow were considered positive staining. For Col II, the results were assessed with the ratio of the relative density of the brown-stained area to the total area (density/total area) [57,58,59]. For MMP-13 and p16 ^Ink4a^, the results were assessed with the immunostaining of positive cells normalized with total cells (positive-stain cell rate) [55,60].

### 2.9. Statistical Analysis

Each in-vitro experiment was repeated at least three times, and data were pooled from the repeated experiments. All data are presented as means ± standard error (means ± SE). For histologic studies, we assessed six fields from the proximal tibia of each guinea pig. In total, there were 48 pictures, chosen from the 8 animals (6 pictures of each guinea pig) analyzed in each group. Comparisons of the data were analyzed using one-way ANOVA, and multiple comparisons were conducted by the LSD posthoc test using SPSS (version 17.1 for Windows; SPSS, Chicago, IL, USA). *P*-value < 0.05 was considered to indicate a statistically significant difference. In mRNA expression, data are the means of three experiments and were analyzed with the use of one-way analysis of variance with Dunnett’s multiple-comparison posthoc test. *P*-values are shown for comparison with controls [61].

## 3. Results

### 3.1. Effect of EGCG on Chondrocyte Viability

**Figure 1:**
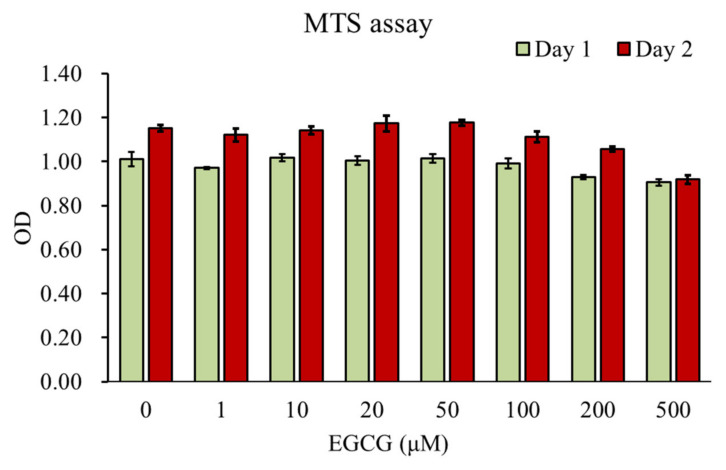
The cell viability of human articular chondrocytes after treatment with EGCG at different concentrations was determined by MTS assay for 24 and 48 h. The columns represent the means ± SE. (*n* = 8, ** *p* < 0.01 vs. the control).

The underlying cytotoxicity of EGCG (concentrations of 1, 10, 20, 50, 100, 200, and 500 μM) on HAC was assessed by MTS assay for 24 and 48 h (Figure 1). The MTS assay showed that EGCG (200 and 500 μM) significantly suppressed cell viability at 24 h (*p* < 0.01 vs. control) and 48 h (*p* < 0.01 vs. control). EGCG showed no cytotoxicity on HAC at concentrations ranging from 0 to 100 μM. In this study, we selected concentrations of 10 μM for the subsequent experiments.

### 3.2. EGCG Suppresses ECM Degradation, Inflammatory Response, Chondrocyte Hypertrophic Differentiation, and Cell Senescence in IL-1β-Stimulated Human Chondrocytes

**Figure 2:**
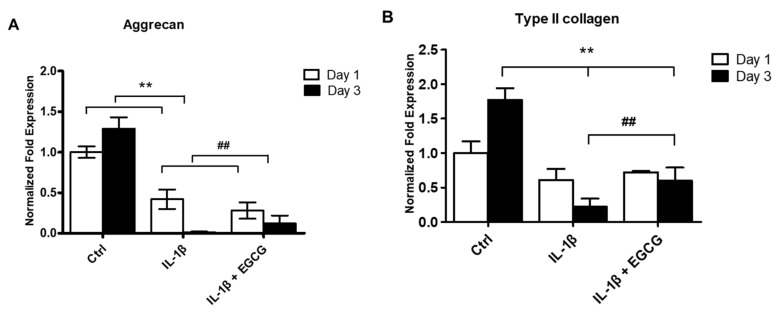

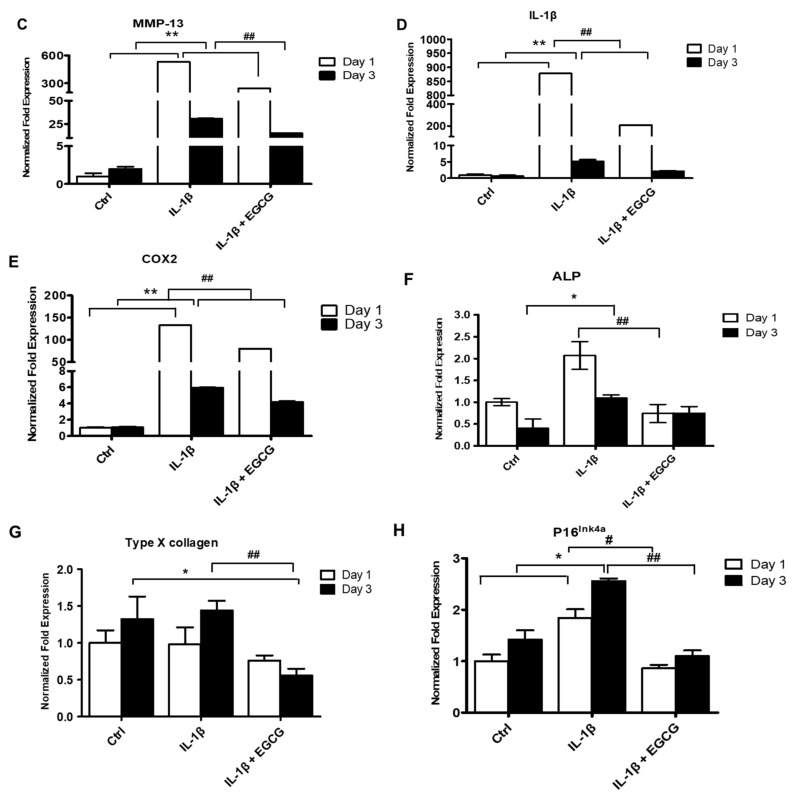
EGCG increased aggrecan and Col II deposition and suppressed MMP-13, IL-1, COX-2, ALP, Col X, p16 ^Ink4a^ expression. Chondrocytes were pretreated with IL-1β (10 ng/mL) for 48 h and then treated with EGCG (10 μM). The cells were collected at 24 and 72 h after EGCG treatment, and aggrecan, Col II, MMP-13, IL-1, COX-2, ALP, Col X, and p16 ^Ink4a^ mRNA expression levels were evaluated by RT-qPCR. (**A**,**B**) The mRNA expression of aggrecan (**A**) and Col II (**B**) were significantly suppressed after IL-1β stimulation and rescued after EGCG treatment at 72 h. (**C**–**H**) The mRNA expression of MMP-13 (**C**), IL-1 (**D**), COX-2 (**E**), ALP (**F**), Col X (**G**), and p16 ^Ink4a^ (**H**) were significantly increased after IL-1β stimulation and reduced after EGCG treatment. The experiments were repeated at least three times, with similar results. All data are represented as the mean ± SE (n = 8 in each group; * *p* < 0.05 and ** *p* < 0.01 versus the control group; # *p* < 0.05 and ## *p* < 0.01 versus the IL-1β-treated group).

IL-1β serves as a potent inducer for cartilage degradation and joint inflammation. Therefore, we investigated by RT-qPCR whether EGCG could reduce the IL-1β-induced inflammatory response, chondrocyte terminal differentiation, ECM degradation, and cell senescence of chondrocytes at 24 and 72 h after EGCG treatment. The results showed that EGCG markedly rescued the IL-1β-induced reduced expression of Col II and aggrecan (Figure 2A,B). Additionally, EGCG significantly reduced the expression of IL-1, COX-2, MMP-13, ALP, Col X, and p16 ^Ink4a^ after IL-1β stimulation at 24 and 72 h (Figure 2C–G).

### 3.3. EGCG Attenuates SAβGal Activity in IL-1β-Stimulated Human Chondrocytes

**Figure 3:**
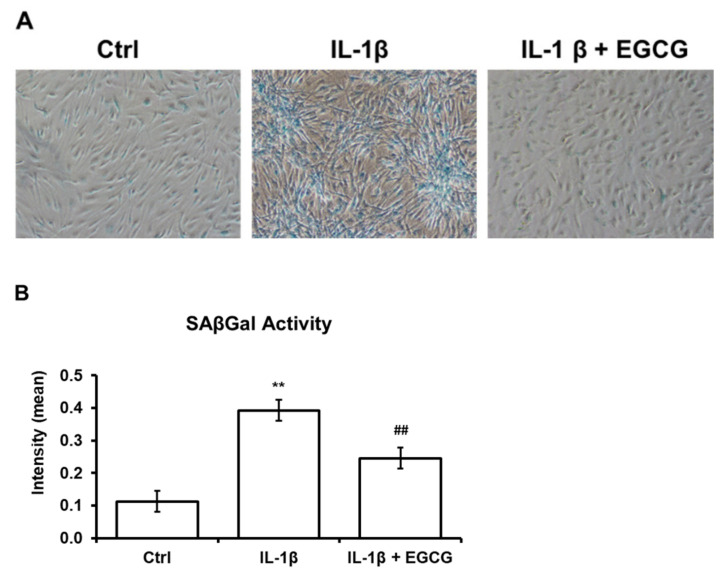
IL-1β induces SAβGal activity in cultured human articular chondrocytes. (**A**) Morphology and SaβGal activity in control, IL-1β-stimulated, and EGCG-treated human articular chondrocytes. A single cell stained blue indicates SAβGal activity. IL-1β-stimulated cells showed a marked increase in SAβGal activity after 48 h. SAβGal activity was significantly reduced after EGCG treatment. (**B**) Quantification of the intensity of SaβGal-stained cells. The intensity of SAβGal activity was significantly enhanced after IL-1β stimulation but significantly reduced while cocultured with EGCG (n = 6 in each group; ** *p* < 0.01 versus the control group; ## *p* < 0.01 versus the IL-1β-treated group).

We used the SAβGal activity level to determine the process of cellular senescence. The average value of SAβGal activity in IL-1β-stimulated HAC was 0.39 ± 0.03, which was 3-fold higher than control (0.11 ± 0.03, *p* < 0.01). EGCG treatment significantly reduced SAβGal activity compared to IL-1β-stimulated chondrocytes (Figure 3).

### 3.4. Intra-Articular Injection of EGCG Improves Running Endurance of OA Animals

**Figure 4:**
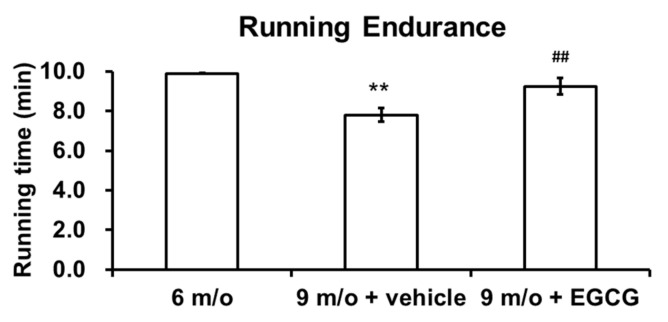
The results of the running endurance test. The running endurance was compared among the animals at 6 months of age (6 m/o), at 9 months of age treated with PBS (9 m/o + vehicle), and at 9 months of age treated with EGCG (9 m/o + EGCG). Running endurance was significantly decreased with age; there was a significant reduction in the running endurance of guinea pigs treated with PBS at 9 months of age compared with animals at 6 months of age. The average running endurance was 9.2 min in the EGCG-treated guinea pigs, which was no different from the endurance of younger guinea pigs (6 months old) and significantly better than that of the same-age animals treated with PBS (*n* = 8 in each group; ** *p* < 0.01 versus animals at 6 months of age; ## *p* < 0.01 versus the animal at 9 months of age, treated with PBS).

All guinea pigs underwent running endurance tests before the first injection at 6 months of age and one week after finishing the last injection at 9 months of age. Measurements were performed three times, and the average data were calculated. As shown in Figure 4, the running endurance significantly decreased with age. The guinea pigs could endure 9.9 min of running at 6 months of age, whereas the guinea pigs could only endure 7.8 min at 9 months of age (*p* < 0.01). Meanwhile, EGCG-treated guinea pigs could endure 9.2 min of running, which was a significant improvement compared to the controlled animals at 9 months of age (*p* < 0.01).

### 3.5. EGCG Treatment Ameliorates Cartilage Degradation

**Figure 5:**
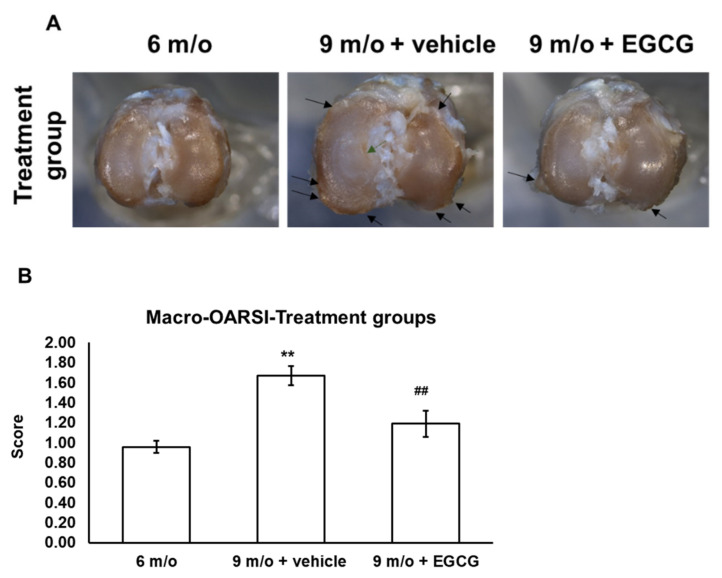
Macroscopic view of right tibial plateau of samples (**A**) and OARSI macroscopic scoring (**B**) between groups, including 6-month-old animals, 9-month-old animals treated with PBS, and 9-month-old animals treated with EGCG. The cartilage surface (especially the medial tibial plateau) of animals became rougher, and there was more severe erosion in the cartilage samples at 9 months of age. Osteophyte formation over the margin of the tibial plateau was also observed in the cartilage samples at 9 months of age than was noted found in the cartilage sampled at 6 months of age. There was less cartilage erosion and osteophyte formation and lower macro-OARSI scores after EGCG treatment (*n* = 8 in each group; ** *p* < 0.01 versus animals at 6 months of age; ## *p* < 0.01 versus the animal treated with PBS at 9 months of age).

The gross morphology at 6 months of age showed mild cartilage erosion on the central area of the tibial plateau. By 9 months of age, the severity of OA increased and displayed typical OA characteristics, such as cartilage erosion, ulceration, and osteophyte formation in the proximal tibia of the controlled animals. The gross morphology of the EGCG-treated group at 9 months of age displayed less severity of cartilage roughness, ulceration, and osteophyte formation (Figure 5A). The macro-OARSI score also represented significantly less OA severity in the EGCG-treated group compared to the control group at 9 months of age (Figure 5B).

**Figure 6:**
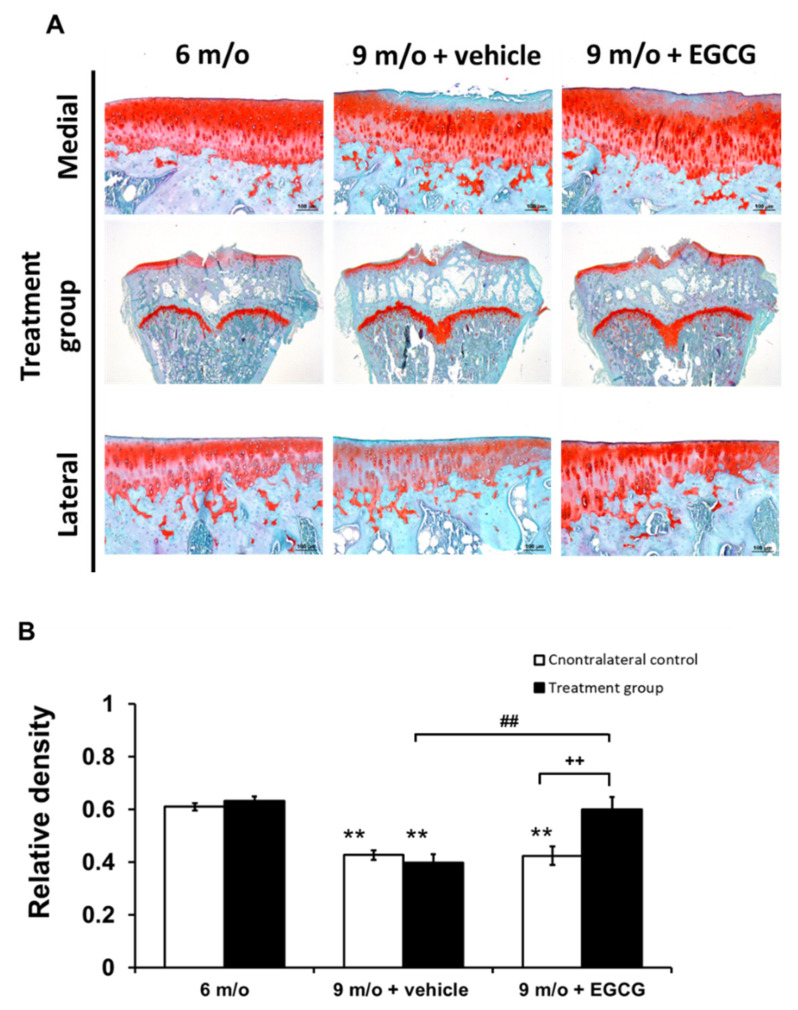
The results of histological analysis of OA and the quantitative assessment of glycosaminoglycan (GAG) loss. (**A**). The representative micrographs of the proximal tibial cartilage with safranin O–Fast Green staining. (**B**). The 9-month-old animals and the cartilage degrading features were decreased after EGCG treatment. Moreover, there was significantly better GAG preservation in the EGCG-treated knee compared with the contralateral untreated knee of animals at 9 months of age (*n* = 8 in each group; ** *p* < 0.01 versus animals at 6 months of age; ## *p* < 0.01 versus animals treated with PBS at 9 months of age; ++ *p* < 0.01 versus the contralateral knee).

Histology further confirmed the macroscopic finding. With the safranin O–Fast Green stain, the articular cartilage of 6-month-old guinea pigs displayed a smooth surface on the medial and lateral tibial plateau. In contrast with the medial plateau, a lesser degree of proteoglycan loss was observed at the lateral compartment. With increasing age, the 9-month-old animals in the control group showed more surface fissures and fibrillation and more proteoglycan loss compared to 6-month-old animals. The cartilage samples of the EGCG-treated animals showed obvious differences compared to the knee joints of controlled animals at 9 months of age, with less articular surface erosion and proteoglycan loss (Figure 6A). As shown in Figure 6, the relative density of stained GAGs in the controlled animals at 9 months of age was significantly lower than animals at 6 months of age (*p* < 0.01). Intra-articular EGCG injection attenuated the cartilage degradation, presenting with less GAG loss compared with the knee joints of the contralateral untreated knee and the controlled animals at 9 months of age (Figure 6B).

These results were further confirmed by the OARSI score, which represents the histopathological changes of cartilage degeneration. The OARSI scores significantly increased with age (6-month-old vs. 9-month-old controls, *p* < 0.01). EGCG treatment significantly reduced the OARSI scores for articular cartilage structure and proteoglycan content. EGCG treatment alleviated cartilage degradation and presented markedly lower OARSI scores (4.16 ± 0.49) compared with controlled animals (5.11 ± 0.49) at 9 months of age (Table 2).

### 3.6. EGCG Maintains the Content of Col II and Attenuates the Expression of MMP-13 and p16 ^Ink4a^ in OA Cartilage

**Figure 7:**
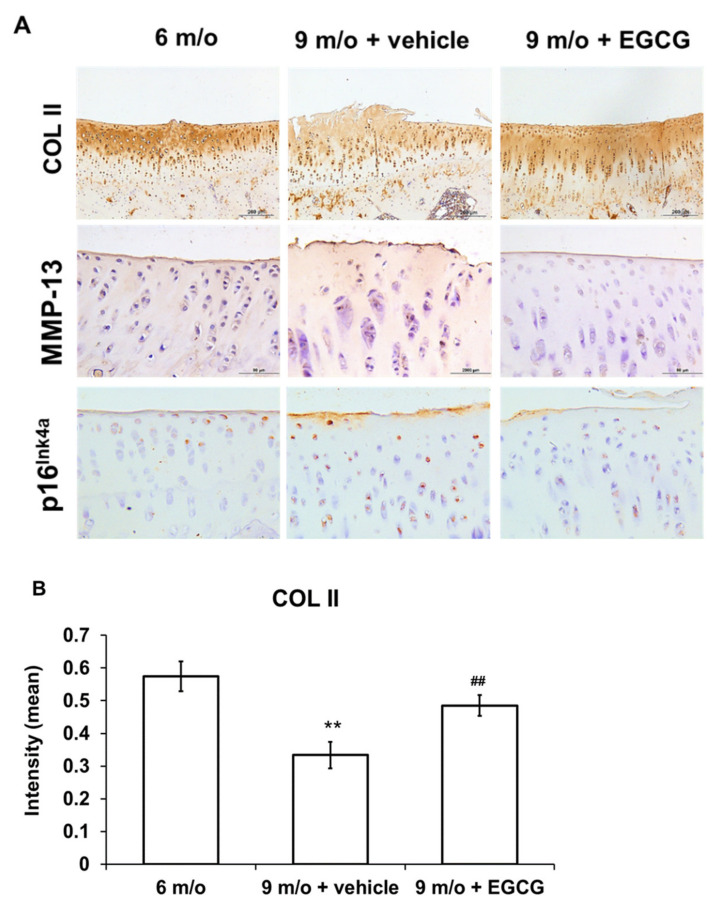

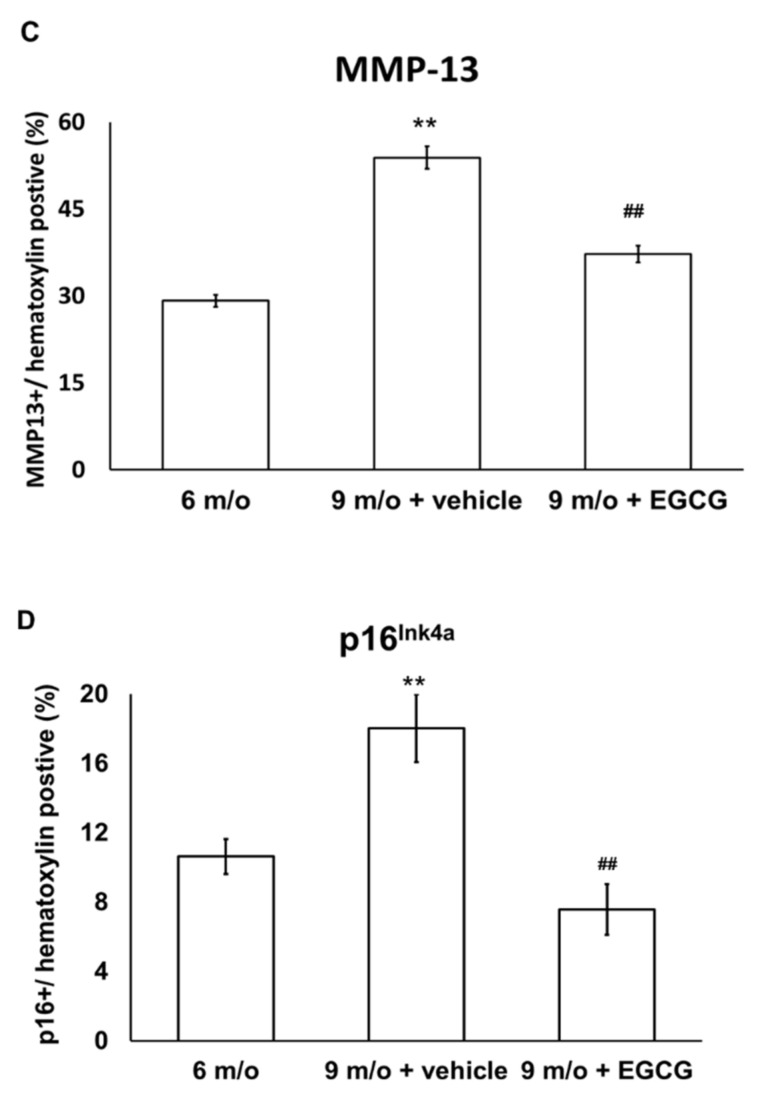
Immunohistochemistry (IHC) of Col II, MMP-13, and p16 ^Ink4a^ in the cartilage samples from animals treated with PBS at 6 months of age and 9 months of age and animals treated with EGCG at 9 months of age. (**A**). The representative micrographs of Col II, MMP-13, and p16 ^Ink4a^ of the proximal tibia of three groups. The intensity of stained Col II and the positive-stained cell ratio of MMP-13 and p16 ^Ink4a^ proteins were measured and compared among groups. The results of Col II are shown in (**B**), MMP-13 in (**C**), and p16 ^Ink4a^ in (**D**) (*n* = 8 in each group; ** *p* < 0.01 versus animals at 6 months of age; ## *p* < 0.01 versus animals treated with PBS at 9 months of age).

We used IHC staining to investigate whether EGCG treatment attenuates OA progression by reducing ECM degradation. The results showed that Col II protein markedly decreased with aging but was elevated after EGCG treatment (Figure 7A). The results revealed that the immunostained MMP-13 protein increased with aging and the treatment of EGCG decreased the staining of MMP-13 compared with the control group at 9 months of age (Figure 7A). The results of the quantitative analysis of Col II and MMP-13 are shown in Figure 7B,C.

Furthermore, immunostained p16 ^Ink4a^ increased significantly at 9 months of age compared to the guinea pigs at 6 months of age but was significantly decreased after EGCG treatment (Figure 7A). Quantitative analysis of the IHC staining of p16 ^Ink4a^ is shown in Figure 7D and demonstrates that EGCG treatment significantly decreased p16 ^Ink4a^ protein expression, which was markedly increased in the knee samples of controlled animals at 9 months of age.

## 4. Discussion

OA is a highly prevalent joint disease that affects 240 million people globally [62]. The prevalence is increasing because of the increasing incidence of OA risk factors, including the rising prevalence of obesity and the aging of populations [63]. The high prevalence of OA entails a substantial economic burden to society, where the years living with the disability of OA is estimated to be more than 12 million [62]. However, there is currently no effective pharmaceutical therapies to attenuate the course of OA progression [64]. EGCG is considered as a potential therapeutic agent for OA because of its potent anti-inflammatory and antioxidative properties [30]. Besides previous health effects, in this study, we further found that the levels of p16 ^Ink4a^ gene expression and SAβGal activity were markedly decreased in IL-1β-stimulated HAC after EGCG treatment, indicating EGCG may attenuate chondrocyte cell senescence after IL-1β stimulation. We also provide direct evidence in the aging-related, naturally occurring OA model of guinea pigs that intra-articular injections of EGCG slow the progression of aging-related cartilage degradation and decrease p16 ^Ink4a^ protein levels in IHC staining.

In this study, we found that the cotreatment of IL-1β-stimulated chondrocytes with EGCG can decrease chondrocyte senescence, as evidenced by a decrease in the gene expression of p16 ^Ink4a^ and the senescence-associated β-galactosidase activity that was upregulated by IL-1β stimulation. IL-1β, a well-known proinflammatory cytokine, is an important pathogenic factor in cartilage degradation and joint inflammation. The chondrocyte is the main cellular target for IL-1β; an upregulation of the type I IL-1 receptor in the chondrocyte was noted in OA cartilage [65]. Human chondrocytes from older adults have been shown to secret more MMP-13 in response to the stimulation of IL-1β [66]. Not only can it induce the secretion of proinflammatory mediators, IL-1β can also stimulate articular chondrocytes to produce reactive oxygen species (ROS) to induce chondrocyte senescence or apoptosis [67,68]. Excessive oxidative stress caused by aging, excess mechanical loading, and inflammatory cytokine stimulation have been found to predispose the development of chondrocyte senescence in OA [69]. Oxidative-stress-induced telomere shortening was more apparent in degenerative cartilage compared with a healthy region that was compatible with histological findings [70]. Mitochondrial dysfunction and reduced activity of the mitochondrial superoxide dismutase are also associated with the imbalanced production of ROS in OA chondrocytes with age [71]. Consequently, excess oxidative stress results in chondrocyte senescence, leading to cartilage degradation and chondrocyte death. EGCG is a potent antioxidant polyphenol that may reduce ROS activity in aging chondrocytes and decrease cell senescence. This hypothesis was further confirmed by an in-vivo study, where an immunostained senescence marker, p16 ^Ink4a^, was significantly reduced after EGCG treatment in aging guinea pigs, accompanied by decrease cartilage degradation and ECM loss.

Age-related inflammation is a significant contributing factor to the development of OA in elderly patients [38]. The reduction of capacity to cope with continuous antigenic load or stress results in a progressive increase in proinflammatory status that occurs with increasing age [72]. Epidemiologic studies have also shown that higher levels of C-reactive protein and IL-6 were found in patients with knee OA and that the level of these inflammatory markers predicted disease progression [73,74]. Elevated senescence markers, such as accumulation of p16 ^ink4a^, telomere attrition, and SAβGal activity, were found in OA chondrocytes [39]. In accordance with previous studies in aging guinea pigs, the density of immunostained MMP-13 protein in articular cartilage increased with age, indicating increasing age-related inflammation [75]. Depleting the p16 ^Ink4a^-expressing senescent cells, both life-long and in late-life, selectively delayed age-related pathologies in muscle cells [76]. Moreover, removing senescent chondrocytes ameliorates OA [40]. With the clearance of senescent chondrocytes expressing p16 ^Ink4a^, the expression of SAβGal activity also decreased [40]. Local clearance of senescent chondrocytes expressing p16 ^Ink4a^ ameliorates post-traumatic OA and further creates a proregenerative environment. Our results indicated that EGCG reduced the inflammatory response in the senescence-associated secretory phenotype (SASP) [77]; IL-1β stimulated human chondrocytes and aging-related reactions.

The chondroprotective effects of EGCG by its anti-inflammatory property have been reported in various models of animal studies. Haqqi et al. first indicated that increased green tea polyphenol consumption reduced the incidence of arthritis as well as the arthritis index compared with mice without green tea polyphenols in water using a collagen-induced arthritis model in mice [78]. Their results showed that increasing the oral intake of green tea polyphenols can significantly reduce joint inflammation, as evidenced by a reduction in the gene expression of inflammatory mediators (COX-2, interferon -γ, and TNF-α), a lesser degree of cell infiltration and vascular pannus formation, as well as a lower level of neutral endopeptidase activity and Col II-specific IgG in arthritic joints [78]. Leong et al. reported a reduction of cartilage degradation of Col II and aggrecan, as well as a decrease in the expression of catabolic genes (MMP-1, -3, -8, and -13, ADAMTS5, IL-1β, and TNF-α) in a surgically induced post-traumatic OA model by destabilization of the medial meniscus in mice [29]. In another study using an intra-articular carrageenan-induced OA model in mice, compared with control, the green tea extract treatment reduced the concentrations of lipid peroxide, nitric oxide, and total thiols in plasma, as well as the presence of the inflammatory, infiltrating cell in synovial tissues [79]. In accordance with these studies, we also found that EGCG could attenuate cartilage degradation in experimental OA. However, the OA model in this study was different from previous research. In the present study, the naturally occurring OA model in guinea pigs was established to mimic the cartilage degenerative process in primary OA, which accounts for the most common cause of OA in the real world. Intra-articular EGCG treatment can attenuate cartilage destruction, decrease the loss of GAG, and alleviate OA symptoms with increasing running endurance, which suggests the antiaging effects of EGCG on primary OA in aged guinea pigs.

There are some limitations to this study. First, we did not use power studies to calculate the sample size in this study. With reference to past research data, 6 to 8 animals are often used to study the effects of therapeutic drugs on degenerative arthritis, so we selected 8 guinea pigs for this study. Secondly, though SA-β-gal is a standard assay for cellular aging, the new staining procedure SenTraGor gives reliable results for senescent cells even in confluent cultures [80]. Further staining with SenTraGor may provide stronger evidence.

## 5. Conclusions

In conclusion, our findings show the anti-OA effect of EGCG in vitro and in vivo. We have shown that EGCG reduces ECM degradation, the inflammatory response, and cell senescence in IL-1β-stimulated human chondrocytes as well as reduces cartilage destruction and ECM degradation in an aging-related OA model. Remarkably, this beneficial action on cartilage is possibly via the inhibition of cell senescence and age-related inflammation. These findings indicate that EGCG could be a potential agent for the treatment of aging-related OA.

## Figures and Tables

**Table 1:** Primer sequences and cycling conditions for real-time PCR.

Human Gene	PCR Primers Sequence (Forward and Reverse)	Annealing Temp. (°C)
Collagen type IIA1 (81bp product)	Forward: 5′-CAA CAC TGC CAA CGT CCA GAT-3′	61
	Reverse: 5′-TCT TGC AGT GGT AGG TGA TGT TCT-3′	
Aggrecan (189bp product)	Forward: 5′-ACA GCT GGG GAC ATT AGT GG -3′	61
	Reverse: 5′-GTG GAA TGC AGA GGT GGT TT-3′	
MMP-13 (161bp product)	Forward:5′-CTT CCC AAC CGT ATT GAT GCT-3′	61
	Reverse: 5′-CTG GTT TCC TGA GAA CAG GAG-3′	
IL-1β (219 bp product)	Forward:5′-GCA ATG AGG ATG ACT TGT TCT-3′	61
	Reverse: 5′-GGT CAT TCT CCT GGA AGG TCT-3′	
PTGS2(COX-2) (157 bp product)	Forward:5′-TGA GCA TCT ACG GTT TGC TG-3′	61
	Reverse: 5′-TGC TTG TCT GGA ACA ACT GC-3′	
Collagen type XA1 (85bp product)	Forward: 5′-AGC CAG GGT TGC CAG GAC CA-3′	61
	Reverse: 5′-TTT TCC CAC TCC AGG AGG GC-3′	
ALP (64bp product)	Forward: 5′-AAC TTC CAG ACC ATT GGC TTG A-3′	64
	Reverse: 5′-TTG CCG CGT GTC GTG TT-3′	
P16 ^Ink4a^ (134bp product)	Forward: 5′-CCAGAGGCAGTAACCATGCC-3′	61
	Reverse: 5′- TTGTGGCCCTGTAGGACCTTC -3′	
GAPDH	Forward: 5′-TCT CCT CTG ACT TCA ACA GCG AC-3′	61
	Reverse: 5′-CCC TGT TGC TGT AGC CAA ATT C-3′	
Cycling conditions	Denature:95 °C for 30 s, 95 °C for 4 min, followed by 35 cycles of 95 °C for 10 s, 58.4–61.5 °C (shown in column of Annealing Temp.) for 15 s and 72 °C for 15 s.	

**Table 2:** OARSI scores.

OARSI Scoring	6 m/o	9 m/o + Vehicle	9m/o + EGCG
Articular cartilage structure	0.90 ± 0.10	1.64 ± 0.13 *	0.92 ± 0.10 ^#^
Proteoglycan content	0.95 ± 0.11	1.4 ± 0.18 *	0.94 ± 0.11 ^#^
cellularity	1.39 ± 0.06	1.65 ± 0.09	1.91 ± 0.10
Tidemark integrity	0.29 ± 0.10	0.32 ± 0.12	0.23 ± 0.09
Additional features osteophyte	0.07 ± 0.01	0.06 ± 0.02	0.16 ± 0.09
Total	3.6 ± 0.39	5.11 ± 0.53 **	4.16 ± 0.49 ^##^

Note: *n* = 8 in each group; * *p* < 0.05 and ** *p* < 0.01 versus animals at 6 months of age; # *p* < 0.05 and ## *p* < 0.01 versus animals treated with PBS at 9 months of age).

## Data Availability

The data presented in this study are available on request from the corresponding author.

## Abbreviations

OA	osteoarthritis
EGCG	(−)-Epigallocatechin 3-gallate
COX-2	cyclooxygenase 2
MMP	matrix metalloproteinase
TNF-α	tumor necrosis factor-α
ADAMTS	a disintegrin and metalloproteinase with thrombospondin motifs
Col II	collagen type II
Col X	collagen X
ECM	extracellular matrix
DH	Dunkin–Hartley
SAβGal	senescence-associated beta-galactosidase
IL-1β	interleukin-1β
ALP	alkaline phosphatase
GAG	glycosaminoglycan
